# Synthesis, crystal structure and Hirshfeld surface analysis of (1*H*-benzimidazol-2-yl)(morpholin-4-yl)methane­thione

**DOI:** 10.1107/S2056989022008933

**Published:** 2022-09-08

**Authors:** Lukmonjon Z. Mutalliev, Sirojiddin Abdullaev, Nasiba Pirnazarova, Ibodat Obidova, Kambarali Turgunov, Ubaydullo Yakubov, Jamshid M. Ashurov, Burkhan Zh. Elmuradov, Azimjon A. Mamadrakhimov

**Affiliations:** a Institute of Bioorganic Chemistry, Academy of Sciences of Uzbekistan, M. Ulugbek Str. 83, Tashkent 700125, Uzbekistan; b Turin Polytechnic University in Tashkent, Kichik Khalka yuli str. 17, 100095 Tashkent, Uzbekistan; c S. Yunusov Institute of Chemistry of Plant Substances, Academy of Sciences of Uzbekistan, Mirzo Ulugbek str. 77, Tashkent 100170, Uzbekistan; Vienna University of Technology, Austria

**Keywords:** (1*H*-benzimidazol-2-yl)(morpholin-4-yl)methane­thione, crystal structure, Hirshfeld surface analysis, Wilgerodt–Kindler reaction

## Abstract

The title compound, C_12_H_13_N_3_OS, was synthesized *via* the Willgerodt–Kindler method. The benzimidozole moiety is essentially planar (r.m.s. deviation = 0.0084 Å). The thio­amide group is inclined by 54.80 (14)° to the benzimidazole ring system. The morpholine ring is disordered over two sets of sites [ratio 0.841 (11):0.159 (11)], with chair conformations for both components. In the crystal, mol­ecules are linked into N—H⋯N hydrogen-bonded chains running parallel to the *c* axis. Hirshfeld surface analysis was used to qu­antify the inter­molecular inter­actions.

## Abstract 2

In the crystal of the title compound, mol­ecules are linked by N—H⋯N hydrogen bonds into chains running parallel to the *c* axis.

## 1. Chemical context

Benzimidazole is a biologically important compound and a useful structural motif for designing mol­ecules of biochemical and pharmacological relevance. Numerous studies have confirmed that these mol­ecules are effective against various strains of microorganisms (El Ashry *et al.*, 2016 ▸). Likewise, substituted benzimidazole derivatives possess various bio­logical activities, including anti­bacterial (Kazimierczuk *et al.*, 2002 ▸), anti­fungal (Ansari & Lal, 2009 ▸), anti­nematode (Mavrova *et al.*, 2006 ▸), anti­viral (Pandey & Shukla, 1999 ▸), anti­cancer (Hranjec *et al.*, 2011 ▸) and anti­protozoal (Mavrova *et al.*, 2010 ▸) properties. Similarly, the morpholine moiety is a versatile and readily accessible synthetic building block; it is easily introduced as an amine reagent or can be built according to a variety of available synthetic methodologies. This versatile scaffold, appropriately substituted, possesses a wide range of biological activities (Walia *et al.*, 2011 ▸). Additionally, most drugs containing a morpholine moiety in their structure have been found to exhibit significant biological properties (Basavaraja *et al.*, 2010 ▸). 
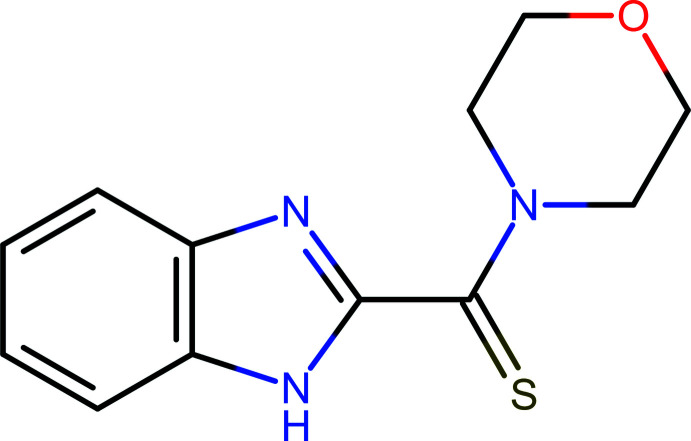


In this context, the title compound with its bifunctional properties (benzimidazole and morpholine derivative, respectively) was synthesized and structurally characterized. The bifunctional properties predispose its potential biological activity, and the three nitro­gen and one sulfur atoms can be used in reactions as electrophilic or nucleophilic sites for the formation of heterocyclic compounds.

## 2. Structural commentary

**Figure 1:**
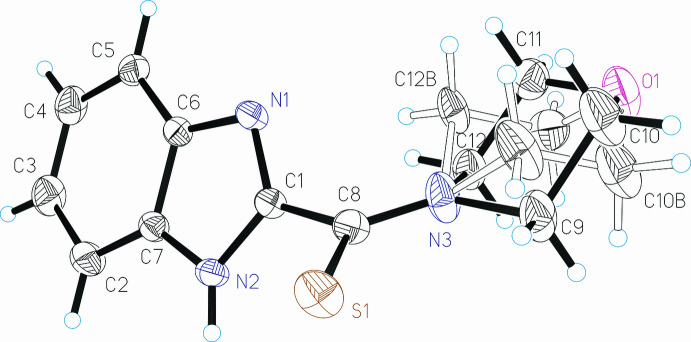
The mol­ecular structure of the title compound with displacement ellipsoids drawn at the 50% probability level. Open bonds refer to the minor component of the disordered morpholide ring.

The title compound crystallizes with one mol­ecule in the asymmetric unit (Fig. 1 ▸). The benzimidazole ring system is essentially planar, with a maximum deviation of 0.013 (3) Å for C6 from the mean plane (r.m.s. deviation = 0.0084 Å). The length of the C1—N2 bond is 1.353 (3) Å, slightly shorter than an isolated single C—N bond (1.382 Å; Berno & Gambarotta, 1994 ▸), while that of the C1—N1 bond is 1.322 (3) Å, slightly longer than an isolated C=N double bond (1.281 Å; Schmaunz *et al.*, 2014 ▸), and the N3—C8 bond length of 1.322 (3) Å is the same as that of C1—N1, indicating conjugation of the *p*-orbital electrons over the imidazole ring. The thio­amide group makes a dihedral angle of 54.80 (14)° with the benzimidazole ring system. Both components of the disordered morpholine ring [occupancy ratio 0.841 (11):0.159 (11)] adopt chair conformations. The puckering parameters (Cremer & Pople, 1975 ▸) of the ring (main occupancy component) are *Q* = 0.521 (6) Å, θ = 176.8 (8)°, *φ* = 80 (8)°. Weak intra­molecular C12—H12*A*⋯N1 and C9—H9*B*⋯S1 hydrogen bonds help to consolidate the conformation of the mol­ecule (Table 1 ▸).

## 3. Supra­molecular features

**Figure 2:**
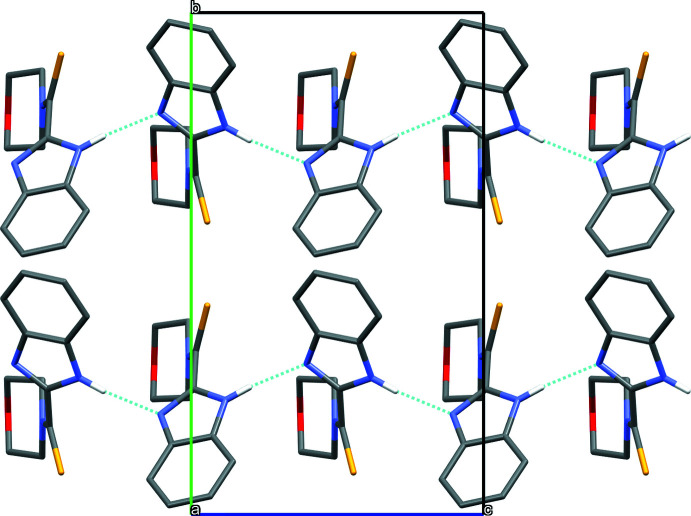
A view of the crystal packing of the title compound along the *a* axis. Inter­molecular N—H⋯N hydrogen bonds are indicated by blue dotted lines. Only the major component of the disordered morpholide ring is shown.

In the crystal, mol­ecules are linked by N2—H2⋯N1 hydrogen bonds into chains running parallel to the *c* axis (Table 1 ▸, Fig. 2 ▸).

**Figure 3:**
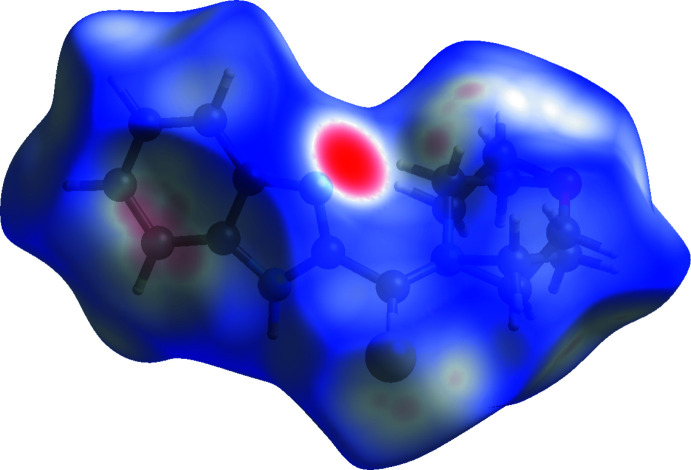
View of the three-dimensional Hirshfeld surface of the title compound plotted over *d*_norm_.

Analysis and calculations of the Hirshfeld surface were carried out with *CrystalExplorer17.5* (Spackman *et al.*, 2021 ▸). The *d*_norm_ plots were mapped with a colour scale between −0.182 a.u. (blue) and 1.195 a.u. (red) and are shown Fig. 3 ▸. The red spots indicate the contribution of N—H⋯N hydrogen bonds.

**Figure 4:**
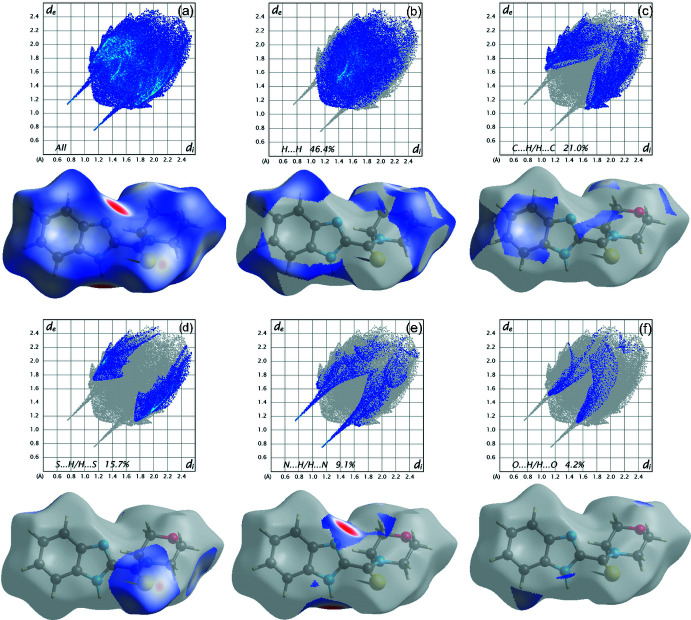
Two-dimensional fingerprint plots of the title compound, showing (*a*) all inter­actions, and delineated into (*b*) H⋯H, (*c*) C⋯H/H⋯C, (*d*) S⋯H/H⋯S, (*e*) N⋯H/H⋯N and (*f*) O⋯H/H⋯O inter­actions.

The expanded two-dimensional fingerprint plots (Seth, 2014 ▸; McKinnon *et al.*, 2007 ▸) are displayed in Fig. 4 ▸ where *d*_e_ and *d*_i_ are the respective distances to the nearest nuclei outside and inside the surface from the Hirshfeld surface. The most important contributions to the crystal packing originate from H⋯H contacts (46.4%), followed by C⋯H/H⋯C contacts (21.0%) and S⋯H/H⋯S contacts (15.7%). Numerical data for other contributions are given in Fig. 4 ▸.

## 4. Database survey

A search in the Cambridge Structural Database (CSD, version 2022; Groom *et al.*, 2016 ▸) gave one match for the benzimidazoyl-thio­carbonate moiety, CSD refcode FUTSOF (Ranskiy *et al.*, 2016 ▸). In the latter compound, the N and S atoms are bound to a Cu^II^ cation. The corresponding N—C bond lengths within the benzimidazole ring exhibit little difference from those the of title compound, except that the C8—S1 bond length is slightly longer [1.708 (7) Å] than in the title compound [1.658 (3) Å]. Another search in the CSD for the morpholin-4-yl-thio­carbonate moiety gave 54 hits, with atomic coordinates not available for five of these structures. In all of the structures, the morpholine ring has a chair conformation, with three structures showing disorder of the morpholine ring [CSD refcodes: QOVVUT (Ramasamy *et al.*, 2009 ▸), TACVIE (Bocheńska *et al.*, 2010 ▸) and YABDAG (Pudovik *et al.*, 1990 ▸)].

## 5. Synthesis and crystallization

**Figure 5:**
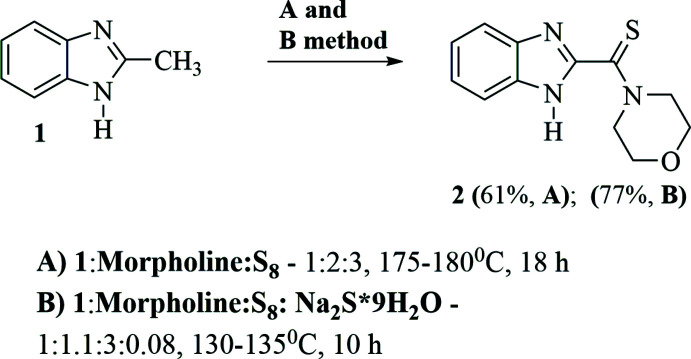
Schematic synthesis of 1*H*-benzimidazol-2-yl(morpholin-4-yl)methane­thione (**2**).

1*H*-Benzimidazol-2-yl(morpholin-4-yl)methane­thione was synthesized using a previously reported procedure with minor modifications (Klingele & Brooker, 2004 ▸; Okamoto *et al.*, 2007 ▸), as shown in Fig. 5 ▸.

Method (i): A reaction mixture consisting of 1.32 g (10 mmol) of 2-methyl­benzimidazole (**1**), 1.68 ml (1.7 g, *d* = 1.01 g ml^−1^, 20 mmol) of morpholine and 0.96 g (30 mmol) of sulfur was heated in a round-bottomed flask at 448–453 K for 18 h. The excess of morpholine was evaporated, and the residue was treated with methanol. The resulting solid was filtered off and recrystallized from benzene, resulting in 1.52 g (61%) of morpholide (**2**). Melting point 513–515 K, *R*_f_ = 0.25 (benzene:acetone 3:1 *v*:*v*).

Method (ii): 1.32 g (10 mmol) of 2-methyl­benzimidazole, 0.92 ml (0.93 g, *d* = 1.01 g ml^−1^, 11.0 mmol) of morpholine, 0.96 g (30 mmol) of sulfur, 0.11 g (0.46 mmol) Na_2_S·9H_2_O and 5 ml of DMSO were mixed and heated in an oil bath at 403–408 K for 10 h. The reaction mixture was cooled to 343 K and extracted three times with 30 ml of a 5%_wt_ NaOH solution. The extracts were combined and filtered. The filtrate was adjusted to pH 5–6 with H_2_SO_4_. The precipitate was filtered off and dried, then recrystallized from benzene and dried again. Yield 1.91 g (77.0%). Melting point 513–515 K, *R*_f_ = 0.25 (benzene:acetone 3:1 *v*:*v*).

^1^H NMR (400 MHz, DMSO-*d*_6_): 12.9 (1H, *s*, NH), 7.7 (1H, *d*, *J* = 8.0, H-4), 7.54 (1H, *d*, *J* = 7.9, H-7), 7.24–7.33 (2H, *m*, H-5,6), 4.37 (2H, *br.t*., *J* = 4.7, NCH_2_-morpholine), 4.22 (2H, *br.t*., *J* = 4.7, NCH_2_-morpholine), 3.82 (2H, *br.t*., *J* = 4.9, OCH_2_-morpholine), 3.71 (2H, *br.t*., *J* = 4.8, OCH_2_-morpholine). ^13^C NMR (400 MHz, DMSO-*d*_6_): 50.19 (NCH_2_-morpholine), 52.95 (NCH_2_-morpholine), 65.94 (OCH_2_-morpholine), 66.62 (OCH_2_-morpholine), 112.2 (C-3a), 120.06 (C-4), 121.3 (C-5), 122.6 (C-6), 124.0 (C-7), 133.9 (C-7a), 142.2 (C-2), 148.9 (C=S). IR (ν, cm^−1^): 1614 (C=N), 1377 (C=S).

A single crystal suitable for X-ray diffraction was selected from crystals obtained by method (ii).

## 6. Refinement

Crystal data, data collection and structure refinement details are summarized in Table 2 ▸. Refinement of the structure with an ordered model gave remaining electron difference peaks about 0.5, 0.26 and 0.24 e^−^ Å^−3^ near the morpholide ring, resulting in *R*1[*F*_o_ > 4σ(*F*_o_)] = 0.039. Introduction of a disorder model including split positions for C9, C10, C11 and C12 of the morpholide ring resulted in a occupancy ratio of 0.841 (11):0.159 (11) for the major and minor components (atoms of the minor component denoted by the *B*). For atom pair C10/C10*B*, the *SHELXL* command EADP was used. All C-bound H atoms were positioned geometrically, with C—H = 0.96 Å (for methyl­ene H atoms) and C—H = 0.93 Å (for aromatic H atoms), and were refined with *U*_iso_(H) = 1.2*U*_eq_(C). The H atom bound to N2 was located in a difference-Fourier map, and its coordinates and isotropic displacement parameter refined freely.

## Supplementary Material

Crystal structure: contains datablock(s) I. DOI: 10.1107/S2056989022008933/wm5654sup1.cif

Structure factors: contains datablock(s) I. DOI: 10.1107/S2056989022008933/wm5654Isup2.hkl

Supporting information file. DOI: 10.1107/S2056989022008933/wm5654Isup3.cml

CCDC reference: 2165380

Additional supporting information:  crystallographic information; 3D view; checkCIF report

## Figures and Tables

**Table 1:** Hydrogen-bond geometry (Å, °)

*D*—H⋯*A*	*D*—H	H⋯*A*	*D*⋯*A*	*D*—H⋯*A*
N2—H2⋯N1^i^	0.84 (4)	2.07 (4)	2.903 (3)	169 (3)
C9—H9*B*⋯S1	0.97	2.60	3.070 (5)	110
C12—H12*A*⋯N1	0.97	2.48	3.131 (5)	124

Symmetry code: (i) 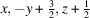 .

**Table 2:** Experimental details

Crystal data	
Chemical formula	C_12_H_13_N_3_OS
*M*_r_	247.31
Crystal system, space group	Monoclinic, *I* *a*
Temperature (K)	293
*a*, *b*, *c* (Å)	8.1644 (2), 15.9237 (3), 9.6936 (2)
β (°)	106.661 (2)
*V* (Å^3^)	1207.33 (5)
*Z*	4
Radiation type	Cu *K*α
μ (mm^−1^)	2.28
Crystal size (mm)	0.30 × 0.25 × 0.14

Data collection	
Diffractometer	XtaLAB Synergy, Single source at home/near, HyPix3000
Absorption correction	Multi-scan (*CrysAlis PRO*; Rigaku OD, 2020 ▸)
*T*_min_, *T*_max_	0.568, 1.000
No. of measured, independent and observed [*I* > 2σ(*I*)] reflections	5160, 1724, 1692
*R*_int_	0.022
(sin θ/λ)_max_ (Å^−1^)	0.614

Refinement	
*R*[*F* ^2^ > 2σ(*F* ^2^)], *wR*(*F* ^2^), *S*	0.030, 0.079, 1.10
No. of reflections	1724
No. of parameters	189
No. of restraints	2
H-atom treatment	H atoms treated by a mixture of independent and constrained refinement
Δρ_max_, Δρ_min_ (e Å^−3^)	0.17, −0.19
Absolute structure	Flack *x* determined using 531 quotients [(*I* ^+^)−(*I* ^−^)]/[(*I* ^+^)+(*I* ^−^)] (Parsons *et al.*, 2013 ▸)
Absolute structure parameter	−0.001 (13)

Computer programs: *CrysAlis PRO* (Rigaku OD, 2020 ▸), *SHELXT* (Sheldrick, 2015*a* ▸), *SHELXL* (Sheldrick, 2015*b* ▸), *XP* (Siemens, 1994 ▸), *Mercury* (Macrae *et al.* 2020 ▸) and *PLATON* (Spek, 2020 ▸).

## supplementary crystallographic information

### Crystal data

**Table:**

C_12_H_13_N_3_OS	*D*_x_ = 1.361 Mg m^−^^3^
*M**_r_* = 247.31	Melting point: 513(2) K
Monoclinic, *I**a*	Cu *K*α radiation, λ = 1.54184 Å
*a* = 8.1644 (2) Å	Cell parameters from 4375 reflections
*b* = 15.9237 (3) Å	θ = 5.5–71.1°
*c* = 9.6936 (2) Å	µ = 2.28 mm^−^^1^
β = 106.661 (2)°	*T* = 293 K
*V* = 1207.33 (5) Å^3^	Needle, colourless
*Z* = 4	0.30 × 0.25 × 0.14 mm
*F*(000) = 520	

### Data collection

**Table:**

XtaLAB Synergy, Single source at home/near, HyPix3000 diffractometer	1724 independent reflections
Radiation source: micro-focus sealed X-ray tube, PhotonJet (Cu) X-ray Source	1692 reflections with *I* > 2σ(*I*)
Mirror monochromator	*R*_int_ = 0.022
Detector resolution: 10.0000 pixels mm^-1^	θ_max_ = 71.3°, θ_min_ = 5.5°
ω scans	*h* = −9→10
Absorption correction: multi-scan (*CrysAlisPro*; Rigaku OD, 2020)	*k* = −19→19
*T*_min_ = 0.568, *T*_max_ = 1.000	*l* = −9→11
5160 measured reflections	

### Refinement

**Table:**

Refinement on *F*^2^	Hydrogen site location: mixed
Least-squares matrix: full	H atoms treated by a mixture of independent and constrained refinement
*R*[*F*^2^ > 2σ(*F*^2^)] = 0.030	*w* = 1/[σ^2^(*F*_o_^2^) + (0.0448*P*)^2^ + 0.2955*P*] where *P* = (*F*_o_^2^ + 2*F*_c_^2^)/3
*wR*(*F*^2^) = 0.079	(Δ/σ)_max_ < 0.001
*S* = 1.10	Δρ_max_ = 0.17 e Å^−^^3^
1724 reflections	Δρ_min_ = −0.19 e Å^−^^3^
189 parameters	Absolute structure: Flack *x* determined using 531 quotients [(*I*^+^)-(*I*^-^)]/[(*I*^+^)+(*I*^-^)] (Parsons *et al.*, 2013)
2 restraints	Absolute structure parameter: −0.001 (13)

### Special details

**Table:**

Geometry. All esds (except the esd in the dihedral angle between two l.s. planes) are estimated using the full covariance matrix. The cell esds are taken into account individually in the estimation of esds in distances, angles and torsion angles; correlations between esds in cell parameters are only used when they are defined by crystal symmetry. An approximate (isotropic) treatment of cell esds is used for estimating esds involving l.s. planes.

### Fractional atomic coordinates and isotropic or equivalent isotropic displacement parameters (Å^2^)

**Table:**

	*x*	*y*	*z*	*U*_iso_*/*U*_eq_	Occ. (<1)
S1	0.43314 (11)	0.91597 (4)	0.55516 (11)	0.0555 (2)	
O1	0.9333 (3)	0.79690 (17)	0.3720 (4)	0.0726 (8)	
N1	0.3522 (3)	0.69924 (13)	0.3950 (2)	0.0334 (4)	
N2	0.3362 (3)	0.72781 (14)	0.6169 (3)	0.0354 (5)	
H2	0.350 (4)	0.754 (2)	0.695 (4)	0.038 (8)*	
N3	0.6584 (3)	0.81395 (16)	0.4919 (4)	0.0555 (8)	
C1	0.3992 (3)	0.74997 (16)	0.5073 (3)	0.0316 (5)	
C2	0.1490 (4)	0.6052 (2)	0.6418 (4)	0.0490 (7)	
H2B	0.1429	0.6168	0.7342	0.059*	
C3	0.0676 (4)	0.5366 (2)	0.5648 (4)	0.0540 (8)	
H3A	0.0048	0.5012	0.6066	0.065*	
C4	0.0767 (4)	0.51882 (19)	0.4256 (4)	0.0498 (7)	
H4A	0.0210	0.4716	0.3780	0.060*	
C5	0.1663 (3)	0.56963 (17)	0.3576 (3)	0.0397 (6)	
H5A	0.1708	0.5581	0.2647	0.048*	
C6	0.2502 (3)	0.63938 (15)	0.4345 (3)	0.0320 (5)	
C7	0.2407 (3)	0.65587 (16)	0.5736 (3)	0.0337 (5)	
C8	0.5083 (3)	0.82515 (16)	0.5156 (3)	0.0377 (6)	
C9	0.7777 (7)	0.8840 (3)	0.4960 (9)	0.0661 (16)	0.841 (11)
H9A	0.8705	0.8823	0.5848	0.079*	0.841 (11)
H9B	0.7187	0.9373	0.4916	0.079*	0.841 (11)
C10	0.8473 (9)	0.8753 (4)	0.3682 (10)	0.078 (2)	0.841 (11)
H10A	0.7541	0.8790	0.2799	0.093*	0.841 (11)
H10B	0.9263	0.9209	0.3686	0.093*	0.841 (11)
C11	0.8153 (7)	0.7297 (3)	0.3658 (7)	0.0530 (12)	0.841 (11)
H11A	0.8722	0.6764	0.3637	0.064*	0.841 (11)
H11B	0.7213	0.7343	0.2782	0.064*	0.841 (11)
C12	0.7468 (6)	0.7322 (2)	0.4947 (7)	0.0466 (11)	0.841 (11)
H12A	0.6675	0.6863	0.4903	0.056*	0.841 (11)
H12B	0.8397	0.7270	0.5828	0.056*	0.841 (11)
C9B	0.715 (5)	0.8869 (14)	0.398 (5)	0.067 (10)	0.159 (11)
H9C	0.6680	0.8780	0.2957	0.080*	0.159 (11)
H9D	0.6840	0.9425	0.4236	0.080*	0.159 (11)
C10B	0.901 (5)	0.873 (2)	0.447 (5)	0.078 (2)	0.159 (11)
H10C	0.9395	0.8654	0.5504	0.093*	0.159 (11)
H10D	0.9605	0.9211	0.4221	0.093*	0.159 (11)
C11B	0.872 (4)	0.7315 (17)	0.446 (4)	0.057 (7)	0.159 (11)
H11C	0.9141	0.7399	0.5495	0.069*	0.159 (11)
H11D	0.9110	0.6772	0.4231	0.069*	0.159 (11)
C12B	0.688 (4)	0.7353 (11)	0.400 (4)	0.047 (7)	0.159 (11)
H12C	0.6372	0.6845	0.4243	0.057*	0.159 (11)
H12D	0.6445	0.7455	0.2971	0.057*	0.159 (11)

### Atomic displacement parameters (Å^2^)

**Table:**

	*U*^11^	*U*^22^	*U*^33^	*U*^12^	*U*^13^	*U*^23^
S1	0.0713 (5)	0.0311 (3)	0.0723 (5)	0.0025 (3)	0.0340 (4)	−0.0069 (4)
O1	0.0627 (14)	0.0675 (16)	0.104 (2)	−0.0080 (12)	0.0499 (15)	−0.0086 (15)
N1	0.0392 (10)	0.0334 (10)	0.0311 (11)	−0.0016 (8)	0.0157 (9)	−0.0017 (8)
N2	0.0419 (12)	0.0381 (11)	0.0294 (12)	−0.0055 (9)	0.0151 (9)	−0.0052 (10)
N3	0.0524 (15)	0.0346 (13)	0.092 (2)	−0.0092 (10)	0.0407 (16)	−0.0091 (13)
C1	0.0349 (13)	0.0307 (11)	0.0307 (12)	−0.0001 (9)	0.0121 (10)	−0.0004 (9)
C2	0.0532 (17)	0.0567 (16)	0.0425 (16)	−0.0104 (14)	0.0226 (14)	0.0030 (14)
C3	0.0517 (17)	0.0498 (16)	0.064 (2)	−0.0166 (14)	0.0231 (16)	0.0053 (15)
C4	0.0434 (15)	0.0411 (15)	0.065 (2)	−0.0097 (11)	0.0154 (14)	−0.0078 (14)
C5	0.0369 (12)	0.0408 (13)	0.0410 (15)	−0.0029 (10)	0.0105 (11)	−0.0085 (12)
C6	0.0314 (11)	0.0330 (11)	0.0325 (12)	0.0012 (9)	0.0107 (10)	−0.0017 (10)
C7	0.0351 (11)	0.0350 (11)	0.0329 (13)	−0.0020 (10)	0.0127 (10)	−0.0002 (10)
C8	0.0460 (14)	0.0318 (12)	0.0375 (14)	−0.0042 (10)	0.0153 (12)	−0.0009 (10)
C9	0.066 (3)	0.052 (2)	0.094 (5)	−0.027 (2)	0.045 (3)	−0.021 (3)
C10	0.093 (4)	0.057 (2)	0.108 (5)	−0.012 (3)	0.067 (5)	−0.003 (4)
C11	0.047 (3)	0.052 (2)	0.061 (3)	0.0026 (18)	0.017 (2)	−0.007 (2)
C12	0.0397 (19)	0.0437 (19)	0.059 (3)	0.0014 (16)	0.019 (2)	−0.0024 (19)
C9B	0.09 (2)	0.030 (9)	0.11 (3)	0.002 (11)	0.07 (2)	0.009 (14)
C10B	0.093 (4)	0.057 (2)	0.108 (5)	−0.012 (3)	0.067 (5)	−0.003 (4)
C11B	0.049 (14)	0.062 (14)	0.058 (17)	0.015 (10)	0.010 (12)	−0.002 (13)
C12B	0.050 (13)	0.029 (8)	0.08 (2)	−0.005 (8)	0.040 (14)	0.002 (10)

### Geometric parameters (Å, º)

**Table:**

S1—C8	1.658 (3)	C5—C6	1.402 (3)
O1—C10	1.427 (7)	C5—H5A	0.9300
O1—C11	1.430 (5)	C6—C7	1.399 (4)
O1—C11B	1.43 (3)	C9—C10	1.511 (10)
O1—C10B	1.48 (4)	C9—H9A	0.9700
N1—C1	1.322 (3)	C9—H9B	0.9700
N1—C6	1.390 (3)	C10—H10A	0.9700
N2—C1	1.353 (3)	C10—H10B	0.9700
N2—C7	1.382 (3)	C11—C12	1.508 (8)
N2—H2	0.84 (4)	C11—H11A	0.9700
N3—C8	1.322 (3)	C11—H11B	0.9700
N3—C9	1.475 (5)	C12—H12A	0.9700
N3—C12	1.485 (5)	C12—H12B	0.9700
N3—C12B	1.60 (2)	C9B—C10B	1.47 (6)
N3—C9B	1.62 (2)	C9B—H9C	0.9700
C1—C8	1.480 (3)	C9B—H9D	0.9700
C2—C3	1.380 (5)	C10B—H10C	0.9700
C2—C7	1.390 (4)	C10B—H10D	0.9700
C2—H2B	0.9300	C11B—C12B	1.44 (4)
C3—C4	1.401 (5)	C11B—H11C	0.9700
C3—H3A	0.9300	C11B—H11D	0.9700
C4—C5	1.379 (4)	C12B—H12C	0.9700
C4—H4A	0.9300	C12B—H12D	0.9700
			
C10—O1—C11	109.5 (4)	O1—C10—C9	110.9 (6)
C11B—O1—C10B	102 (2)	O1—C10—H10A	109.5
C1—N1—C6	104.3 (2)	C9—C10—H10A	109.5
C1—N2—C7	106.6 (2)	O1—C10—H10B	109.5
C1—N2—H2	127 (2)	C9—C10—H10B	109.5
C7—N2—H2	127 (2)	H10A—C10—H10B	108.0
C8—N3—C9	122.1 (3)	O1—C11—C12	110.5 (4)
C8—N3—C12	125.8 (3)	O1—C11—H11A	109.5
C9—N3—C12	110.4 (3)	C12—C11—H11A	109.5
C8—N3—C12B	120.0 (9)	O1—C11—H11B	109.5
C8—N3—C9B	115.3 (10)	C12—C11—H11B	109.5
C12B—N3—C9B	97.8 (16)	H11A—C11—H11B	108.1
N1—C1—N2	113.7 (2)	N3—C12—C11	107.6 (4)
N1—C1—C8	124.5 (2)	N3—C12—H12A	110.2
N2—C1—C8	121.8 (2)	C11—C12—H12A	110.2
C3—C2—C7	116.4 (3)	N3—C12—H12B	110.2
C3—C2—H2B	121.8	C11—C12—H12B	110.2
C7—C2—H2B	121.8	H12A—C12—H12B	108.5
C2—C3—C4	122.0 (3)	C10B—C9B—N3	99 (3)
C2—C3—H3A	119.0	C10B—C9B—H9C	112.1
C4—C3—H3A	119.0	N3—C9B—H9C	112.1
C5—C4—C3	121.6 (3)	C10B—C9B—H9D	112.1
C5—C4—H4A	119.2	N3—C9B—H9D	112.1
C3—C4—H4A	119.2	H9C—C9B—H9D	109.7
C4—C5—C6	117.2 (3)	C9B—C10B—O1	106 (3)
C4—C5—H5A	121.4	C9B—C10B—H10C	110.5
C6—C5—H5A	121.4	O1—C10B—H10C	110.5
N1—C6—C7	109.9 (2)	C9B—C10B—H10D	110.5
N1—C6—C5	129.6 (2)	O1—C10B—H10D	110.5
C7—C6—C5	120.5 (2)	H10C—C10B—H10D	108.7
N2—C7—C2	132.2 (3)	O1—C11B—C12B	107 (2)
N2—C7—C6	105.4 (2)	O1—C11B—H11C	110.3
C2—C7—C6	122.4 (2)	C12B—C11B—H11C	110.3
N3—C8—C1	117.1 (2)	O1—C11B—H11D	110.3
N3—C8—S1	125.5 (2)	C12B—C11B—H11D	110.3
C1—C8—S1	117.5 (2)	H11C—C11B—H11D	108.5
N3—C9—C10	108.0 (5)	C11B—C12B—N3	100 (3)
N3—C9—H9A	110.1	C11B—C12B—H12C	111.8
C10—C9—H9A	110.1	N3—C12B—H12C	111.8
N3—C9—H9B	110.1	C11B—C12B—H12D	111.8
C10—C9—H9B	110.1	N3—C12B—H12D	111.8
H9A—C9—H9B	108.4	H12C—C12B—H12D	109.5
			
C6—N1—C1—N2	0.1 (3)	C12—N3—C8—S1	162.8 (4)
C6—N1—C1—C8	−179.1 (2)	C12B—N3—C8—S1	−156.9 (15)
C7—N2—C1—N1	0.6 (3)	C9B—N3—C8—S1	−40.3 (19)
C7—N2—C1—C8	179.8 (2)	N1—C1—C8—N3	−55.2 (4)
C7—C2—C3—C4	0.1 (5)	N2—C1—C8—N3	125.7 (3)
C2—C3—C4—C5	−0.7 (5)	N1—C1—C8—S1	125.4 (2)
C3—C4—C5—C6	0.9 (4)	N2—C1—C8—S1	−53.7 (3)
C1—N1—C6—C7	−0.7 (3)	C8—N3—C9—C10	−135.7 (5)
C1—N1—C6—C5	−179.5 (3)	C12—N3—C9—C10	58.2 (9)
C4—C5—C6—N1	178.1 (3)	C11—O1—C10—C9	61.3 (8)
C4—C5—C6—C7	−0.5 (4)	N3—C9—C10—O1	−59.2 (9)
C1—N2—C7—C2	179.1 (3)	C10—O1—C11—C12	−62.0 (8)
C1—N2—C7—C6	−1.0 (3)	C8—N3—C12—C11	135.6 (4)
C3—C2—C7—N2	−179.8 (3)	C9—N3—C12—C11	−58.9 (7)
C3—C2—C7—C6	0.3 (5)	O1—C11—C12—N3	60.2 (6)
N1—C6—C7—N2	1.1 (3)	C8—N3—C9B—C10B	157 (2)
C5—C6—C7—N2	180.0 (2)	C12B—N3—C9B—C10B	−74 (3)
N1—C6—C7—C2	−179.0 (3)	N3—C9B—C10B—O1	76 (3)
C5—C6—C7—C2	−0.1 (4)	C11B—O1—C10B—C9B	−73 (4)
C9—N3—C8—C1	179.5 (5)	C10B—O1—C11B—C12B	74 (4)
C12—N3—C8—C1	−16.6 (5)	O1—C11B—C12B—N3	−77 (3)
C12B—N3—C8—C1	23.7 (15)	C8—N3—C12B—C11B	−160.0 (16)
C9B—N3—C8—C1	140.3 (19)	C9B—N3—C12B—C11B	75 (3)
C9—N3—C8—S1	−1.1 (6)		

### Hydrogen-bond geometry (Å, º)

**Table:**

*D*—H···*A*	*D*—H	H···*A*	*D*···*A*	*D*—H···*A*
N2—H2···N1^i^	0.84 (4)	2.07 (4)	2.903 (3)	169 (3)
C9—H9*B*···S1	0.97	2.60	3.070 (5)	110
C12—H12*A*···N1	0.97	2.48	3.131 (5)	124

Symmetry code: (i) *x*, −*y*+3/2, *z*+1/2.
